# Supplementary material for: Jumps and Cojumps analyses of major and minor cryptocurrencies
Source: PLoS One. 2021 Feb 3;16(2):e0245744. doi: 10.1371/journal.pone.0245744 (PMC7857619; doi:10.1371/journal.pone.0245744)
Supplement: S1 Table — (DOCX) [file pone.0245744.s001.docx]

**S1 Table. List of Cryptocurrencies**

This table presents the list of cryptocurrencies considered in this paper as well as their full name and the market capitalization according to CoinMarketCap (accessed on June, 2020).

| **Cryptocurrencies Ranked by Market Capitalization** | | | |
| --- | --- | --- | --- |
| **Cryptocurrency** | **Full Name** | **Market Cap ($)** | **Obs** |
| BTC | Bitcoin | 222,472,909,975 | 123 |
| ETH | Ethereum | 47,639,865,577 | 123 |
| XRP | XRP | 13,675,694,760 | 123 |
| LINK | Chainlink | 5,769,677,737 | 123 |
| LTC | Litecoin | 4,313,178,927 | 123 |
| ADA | Cardano | 3,549,308,711 | 123 |
| EOS | EOS | 3,383,522,261 | 123 |
| BNB | Binance Coin | 3,319,013,682 | 123 |
| XLM | Stellar | 2,246,175,409 | 123 |
| TRX | TRON | 2,189,426,967 | 123 |
| XMR | Monero | 1,647,287,485 | 123 |
| NEO | Neo | 1,152,261,929 | 123 |
| IOTA | IOTA | 1,080,514,273 | 123 |
| DASH | Dash | 905,612,489 | 123 |
| ETC | Ethereum Classic | 860,013,024 | 123 |
| ZEC | Zcash | 826,343,091 | 123 |
| LEND | Aave | 733,636,238 | 123 |
| BAT | Basic Attention Token | 513,332,449 | 123 |
| WAVES | Waves | 417,090,212 | 123 |
| ZRX | 0x | 396,491,474 | 123 |
| OMG | OMG Network | 366,101,986 | 123 |
| KNC | Kyber Network | 331,637,638 | 123 |
| QTUM | Qtum | 321,279,036 | 123 |
| ICX | ICON | 275,087,252 | 123 |
| LSK | Lisk | 210,146,025 | 123 |
| LRC | Loopring | 202,683,954 | 123 |
| BTG | Bitcoin Gold | 196,176,292 | 123 |
| NANO | Nano | 170,987,184 | 123 |
| ENJ | Enjin Coin | 167,446,534 | 123 |
| BCD | Bitcoin Diamond | 152,161,633 | 123 |
| BNT | Bancor | 136,340,423 | 123 |
| RLC | iExec RLC | 129,019,279 | 123 |
| MANA | Decentraland | 126,940,133 | 123 |
| SNT | Status | 122,614,072 | 123 |
| XVG | Verge | 114,344,226 | 123 |
| IOST | IOST | 110,407,776 | 123 |
| BTS | BitShares | 96,336,963 | 123 |
| KMD | Komodo | 92,430,876 | 123 |
| STEEM | Steem | 90,398,825 | 123 |
| MCO | MCO | 88,260,023 | 123 |
| XZC | Zcoin | 74,627,113 | 123 |
| ELF | aelf | 64,811,039 | 123 |
| ARK | Ark | 63,810,445 | 123 |
| STRAT | Stratis | 59,757,753 | 123 |
| AION | Aion | 55,823,051 | 123 |
| STORJ | Storj | 54,423,108 | 123 |
| WTC | Waltonchain | 45,069,212 | 123 |
| ENG | Enigma | 44,435,923 | 123 |
| POWR | Power Ledger | 44,299,114 | 123 |
| NULS | NULS | 43,259,444 | 123 |
| RCN | Ripio Credit Network | 39,252,762 | 123 |
| AST | AirSwap | 38,386,089 | 123 |
| FUN | FunFair | 36,534,829 | 123 |
| REQ | Request | 36,515,294 | 123 |
