# Supplementary material for: Jumps and Cojumps analyses of major and minor cryptocurrencies
Source: PLoS One. 2021 Feb 3;16(2):e0245744. doi: 10.1371/journal.pone.0245744 (PMC7857619; doi:10.1371/journal.pone.0245744)
Supplement: S2 Table — (DOCX) [file pone.0245744.s002.docx]

**S2 Table: Summary Statistics for Realised Variation**

This table presents statistics that summarize the unconditional distributions of daily (square rooted) realized variation of the SET100 index, major cryptocurrencies and the minor cryptocurrencies. Table A in the appendix presents the list of cryptocurrencies (symbols) considered in this paper as well as their full name and the associated market capitalization according to CoinMarketCap (accessed on June, 2020).

| **SET INDEX** | **Mean** | **Std** | **Kurtosis** | **Skewness** | **Obs** |
| --- | --- | --- | --- | --- | --- |
| SET100 | 0.0073 | 0.0019 | 3.3248 | 1.5371 | 123 |
| **Cryptocurrencies Ranked by Market Capitalization** | | | | | |
| **Cryptocurrency** | **Mean** | **Std** | **Kurtosis** | **Skewness** | **Obs** |
| BTC | 0.0389 | 0.0170 | 1.1743 | 1.2141 | 123 |
| ETH | 0.0260 | 0.0082 | 2.5008 | 1.2518 | 123 |
| XRP | 0.0342 | 0.0160 | 2.4518 | 1.5814 | 123 |
| LINK | 0.0723 | 0.0249 | 7.5802 | 2.0636 | 123 |
| LTC | 0.0310 | 0.0092 | 1.1624 | 1.0487 | 123 |
| ADA | 0.0468 | 0.0187 | 6.1534 | 1.9162 | 123 |
| EOS | 0.0517 | 0.0217 | 1.4883 | 1.2912 | 123 |
| BNB | 0.0480 | 0.0250 | 19.5935 | 3.4495 | 123 |
| XLM | 0.0443 | 0.0192 | 3.3139 | 1.4702 | 123 |
| TRX | 0.0588 | 0.0271 | 10.3692 | 2.4936 | 123 |
| XMR | 0.0440 | 0.0129 | 1.3836 | 1.1329 | 123 |
| NEO | 0.0467 | 0.0175 | 2.3980 | 1.4425 | 123 |
| IOTA | 0.0570 | 0.0239 | 7.4844 | 2.2279 | 123 |
| DASH | 0.0418 | 0.0141 | 8.8589 | 2.2907 | 123 |
| ETC | 0.0450 | 0.0221 | 8.6778 | 2.4416 | 123 |
| ZEC | 0.0526 | 0.0307 | 29.5416 | 4.4973 | 123 |
| LEND | 0.0846 | 0.0239 | 2.6075 | 1.2777 | 123 |
| BAT | 0.0755 | 0.0332 | 10.0817 | 2.8055 | 123 |
| WAVES | 0.0494 | 0.0146 | 0.6811 | 0.8987 | 123 |
| ZRX | 0.0753 | 0.0405 | 19.5035 | 3.8380 | 123 |
| OMG | 0.0532 | 0.0234 | 16.4238 | 3.2182 | 123 |
| KNC | 0.0715 | 0.0511 | 56.8092 | 6.9548 | 123 |
| QTUM | 0.0501 | 0.0198 | 10.9596 | 2.7674 | 123 |
| ICX | 0.0682 | 0.0306 | 4.0968 | 1.7486 | 123 |
| LSK | 0.0525 | 0.0226 | 6.7383 | 2.3135 | 123 |
| LRC | 0.0756 | 0.0381 | 24.0903 | 3.9833 | 123 |
| BTG | 0.0557 | 0.0172 | 1.6100 | 1.3091 | 123 |
| NANO | 0.0727 | 0.0326 | 5.9566 | 1.9984 | 123 |
| ENJ | 0.0712 | 0.0241 | 3.0413 | 1.4335 | 123 |
| BCD | 0.0906 | 0.0564 | 15.1908 | 3.3444 | 123 |
| BNT | 0.0589 | 0.0261 | 60.6612 | 6.6623 | 123 |
| RLC | 0.0845 | 0.0504 | 50.8557 | 6.2399 | 123 |
| MANA | 0.0790 | 0.0662 | 59.3903 | 7.2658 | 123 |
| SNT | 0.0579 | 0.0252 | 6.6338 | 1.9878 | 123 |
| XVG | 0.0752 | 0.0426 | 10.9790 | 2.7814 | 123 |
| IOST | 0.0858 | 0.0361 | 28.5370 | 4.3975 | 123 |
| BTS | 0.0554 | 0.0149 | 9.3742 | 2.3443 | 123 |
| KMD | 0.0754 | 0.0292 | 19.9300 | 3.6748 | 123 |
| STEEM | 0.0751 | 0.0372 | 11.2602 | 2.9147 | 123 |
| MCO | 0.0745 | 0.0555 | 81.9503 | 8.2933 | 123 |
| XZC | 0.0683 | 0.0297 | 31.5855 | 4.5099 | 123 |
| ELF | 0.0696 | 0.0326 | 8.7931 | 2.5489 | 123 |
| ARK | 0.0751 | 0.0240 | 7.3073 | 2.2281 | 123 |
| STRAT | 0.0561 | 0.0176 | 1.4060 | 1.0121 | 123 |
| AION | 0.0848 | 0.0425 | 38.5982 | 4.9966 | 123 |
| STORJ | 0.0728 | 0.0377 | 48.0959 | 5.8025 | 123 |
| WTC | 0.0819 | 0.0278 | 3.1976 | 1.4568 | 123 |
| ENG | 0.0705 | 0.0221 | 3.3282 | 1.5313 | 123 |
| POWR | 0.0696 | 0.0267 | 3.4475 | 1.5953 | 123 |
| NULS | 0.0781 | 0.0276 | 2.4015 | 1.4366 | 123 |
| RCN | 0.0713 | 0.0300 | 7.6105 | 2.3037 | 123 |
| AST | 0.0940 | 0.0519 | 7.1423 | 2.5417 | 123 |
| FUN | 0.0756 | 0.0286 | 17.7036 | 3.3075 | 123 |
| REQ | 0.0819 | 0.0353 | 21.4560 | 3.7049 | 123 |
