# Supplementary material for: Jumps and Cojumps analyses of major and minor cryptocurrencies
Source: PLoS One. 2021 Feb 3;16(2):e0245744. doi: 10.1371/journal.pone.0245744 (PMC7857619; doi:10.1371/journal.pone.0245744)
Supplement: S3 Table — (DOCX) [file pone.0245744.s003.docx]

**S3 Table: Summary Statistics for Continuous Sample Path Variation**

This table presents statistics that summarize the unconditional distributions of daily (square rooted) continuous sample path variation of the SET100 index, major cryptocurrencies and the minor cryptocurrencies. Table A in the appendix presents the list of cryptocurrencies (symbols) considered in this paper as well as their full name and the associated market capitalization according to CoinMarketCap (accessed on June, 2020).

| **SET INDEX** | **Mean** | **Std** | **Kurtosis** | **Skewness** | **Obs** |
| --- | --- | --- | --- | --- | --- |
| SET100 | 0.0072 | 0.0018 | 2.1476 | 1.3342 | 123 |
| **Cryptocurrencies Ranked by Market Capitalization** | | | | | |
| **Cryptocurrency** | **Mean** | **Std** | **Kurtosis** | **Skewness** | **Obs** |
| BTC | 0.0372 | 0.0166 | 1.4869 | 1.2987 | 123 |
| ETH | 0.0255 | 0.0085 | 2.3995 | 1.2763 | 123 |
| XRP | 0.0338 | 0.0161 | 2.3971 | 1.5761 | 123 |
| LINK | 0.0696 | 0.0253 | 7.7830 | 2.1348 | 123 |
| LTC | 0.0302 | 0.0091 | 0.6034 | 0.9252 | 123 |
| ADA | 0.0457 | 0.0183 | 6.6712 | 1.9038 | 123 |
| EOS | 0.0503 | 0.0214 | 1.8559 | 1.3998 | 123 |
| BNB | 0.0474 | 0.0252 | 19.3126 | 3.4283 | 123 |
| XLM | 0.0432 | 0.0187 | 2.0030 | 1.2744 | 123 |
| TRX | 0.0574 | 0.0276 | 9.9964 | 2.4596 | 123 |
| XMR | 0.0423 | 0.0124 | 2.4461 | 1.3808 | 123 |
| NEO | 0.0458 | 0.0174 | 1.9645 | 1.3524 | 123 |
| IOTA | 0.0549 | 0.0219 | 3.1164 | 1.6667 | 123 |
| DASH | 0.0403 | 0.0135 | 9.5473 | 2.2640 | 123 |
| ETC | 0.0439 | 0.0210 | 7.1357 | 2.2718 | 123 |
| ZEC | 0.0510 | 0.0306 | 30.8361 | 4.6315 | 123 |
| LEND | 0.0804 | 0.0244 | 2.8941 | 1.4122 | 123 |
| BAT | 0.0738 | 0.0333 | 10.5402 | 2.8826 | 123 |
| WAVES | 0.0478 | 0.0140 | 0.2560 | 0.7429 | 123 |
| ZRX | 0.0740 | 0.0409 | 19.0648 | 3.7922 | 123 |
| OMG | 0.0512 | 0.0210 | 9.8096 | 2.5949 | 123 |
| KNC | 0.0675 | 0.0487 | 66.7730 | 7.3874 | 123 |
| QTUM | 0.0494 | 0.0198 | 11.4219 | 2.8546 | 123 |
| ICX | 0.0667 | 0.0305 | 4.4758 | 1.8211 | 123 |
| LSK | 0.0509 | 0.0217 | 5.0594 | 2.0808 | 123 |
| LRC | 0.0718 | 0.0324 | 8.2650 | 2.4767 | 123 |
| BTG | 0.0544 | 0.0172 | 1.8981 | 1.3744 | 123 |
| NANO | 0.0711 | 0.0326 | 6.2537 | 2.0277 | 123 |
| ENJ | 0.0686 | 0.0239 | 3.4348 | 1.4716 | 123 |
| BCD | 0.0867 | 0.0572 | 14.9575 | 3.3015 | 123 |
| BNT | 0.0504 | 0.0201 | 22.9390 | 3.4040 | 123 |
| RLC | 0.0799 | 0.0509 | 50.7801 | 6.2634 | 123 |
| MANA | 0.0773 | 0.0665 | 58.9569 | 7.2291 | 123 |
| SNT | 0.0556 | 0.0236 | 5.5806 | 1.8059 | 123 |
| XVG | 0.0726 | 0.0433 | 10.7755 | 2.7868 | 123 |
| IOST | 0.0822 | 0.0316 | 14.3794 | 3.0687 | 123 |
| BTS | 0.0548 | 0.0145 | 8.8961 | 2.2149 | 123 |
| KMD | 0.0717 | 0.0295 | 20.8251 | 3.8221 | 123 |
| STEEM | 0.0722 | 0.0347 | 11.3649 | 2.8679 | 123 |
| MCO | 0.0697 | 0.0381 | 53.8238 | 6.2317 | 123 |
| XZC | 0.0629 | 0.0294 | 36.6173 | 5.0119 | 123 |
| ELF | 0.0670 | 0.0325 | 9.7062 | 2.7036 | 123 |
| ARK | 0.0716 | 0.0216 | 3.7139 | 1.6197 | 123 |
| STRAT | 0.0536 | 0.0159 | 1.9125 | 0.9994 | 123 |
| AION | 0.0803 | 0.0355 | 26.0135 | 3.9145 | 123 |
| STORJ | 0.0684 | 0.0388 | 45.3173 | 5.5988 | 123 |
| WTC | 0.0785 | 0.0266 | 4.3151 | 1.6324 | 123 |
| ENG | 0.0683 | 0.0223 | 3.5591 | 1.5506 | 123 |
| POWR | 0.0667 | 0.0256 | 1.8477 | 1.3408 | 123 |
| NULS | 0.0735 | 0.0268 | 3.5405 | 1.6258 | 123 |
| RCN | 0.0681 | 0.0295 | 9.0432 | 2.5022 | 123 |
| AST | 0.0906 | 0.0526 | 7.1963 | 2.5471 | 123 |
| FUN | 0.0723 | 0.0293 | 17.2364 | 3.2943 | 123 |
| REQ | 0.0780 | 0.0293 | 7.6229 | 2.2677 | 123 |
