# Supplementary material for: Jumps and Cojumps analyses of major and minor cryptocurrencies
Source: PLoS One. 2021 Feb 3;16(2):e0245744. doi: 10.1371/journal.pone.0245744 (PMC7857619; doi:10.1371/journal.pone.0245744)
Supplement: S4 Table — (DOCX) [file pone.0245744.s004.docx]

**S4 Table: Summary Statistics for Jump Variation**

This table presents statistics that summarize the unconditional distributions of daily (square rooted) jump variation of the SET100 index, major cryptocurrencies and the minor cryptocurrencies. Table A in the appendix presents the list of cryptocurrencies (symbols) considered in this paper as well as their full name and the associated market capitalization according to CoinMarketCap (accessed on June, 2020).

| **SET INDEX** | **Mean** | **Std** | **Kurtosis** | **Skewness** | **Obs** |
| --- | --- | --- | --- | --- | --- |
| SET100 | 0.0005 | 0.0015 | 10.8701 | 3.2358 | 123 |
| **Cryptocurrencies Ranked by Market Capitalization** | | | | | |
| **Cryptocurrency** | **Mean** | **Std** | **Kurtosis** | **Skewness** | **Obs** |
| BTC | 0.0062 | 0.0103 | 1.2010 | 1.4959 | 123 |
| ETH | 0.0023 | 0.0045 | 0.9625 | 1.6005 | 123 |
| XRP | 0.0015 | 0.0039 | 6.0402 | 2.6429 | 123 |
| LINK | 0.0113 | 0.0152 | -0.6580 | 0.8693 | 123 |
| LTC | 0.0035 | 0.0066 | 2.4232 | 1.7694 | 123 |
| ADA | 0.0046 | 0.0096 | 7.1872 | 2.5097 | 123 |
| EOS | 0.0057 | 0.0109 | 1.8418 | 1.7226 | 123 |
| BNB | 0.0028 | 0.0069 | 3.7767 | 2.2686 | 123 |
| XLM | 0.0048 | 0.0097 | 6.6252 | 2.4139 | 123 |
| TRX | 0.0058 | 0.0101 | 0.9429 | 1.4629 | 123 |
| XMR | 0.0064 | 0.0106 | 0.6128 | 1.3738 | 123 |
| NEO | 0.0040 | 0.0087 | 11.6001 | 2.8475 | 123 |
| IOTA | 0.0081 | 0.0161 | 25.4274 | 4.0615 | 123 |
| DASH | 0.0061 | 0.0102 | 4.5576 | 1.9757 | 123 |
| ETC | 0.0044 | 0.0114 | 17.3669 | 3.7546 | 123 |
| ZEC | 0.0062 | 0.0117 | 4.8714 | 2.0886 | 123 |
| LEND | 0.0164 | 0.0202 | -1.1031 | 0.6488 | 123 |
| BAT | 0.0073 | 0.0138 | 1.5767 | 1.6594 | 123 |
| WAVES | 0.0066 | 0.0114 | 1.6830 | 1.6041 | 123 |
| ZRX | 0.0060 | 0.0111 | 1.2108 | 1.5935 | 123 |
| OMG | 0.0078 | 0.0159 | 20.6378 | 3.6490 | 123 |
| KNC | 0.0145 | 0.0243 | 46.0362 | 5.5087 | 123 |
| QTUM | 0.0031 | 0.0078 | 5.7407 | 2.5407 | 123 |
| ICX | 0.0056 | 0.0132 | 12.5371 | 3.0552 | 123 |
| LSK | 0.0067 | 0.0126 | 10.0826 | 2.6702 | 123 |
| LRC | 0.0132 | 0.0280 | 48.5836 | 5.8992 | 123 |
| BTG | 0.0051 | 0.0110 | 5.0694 | 2.2739 | 123 |
| NANO | 0.0069 | 0.0139 | 3.8787 | 2.0858 | 123 |
| ENJ | 0.0107 | 0.0162 | 0.7584 | 1.2939 | 123 |
| BCD | 0.0149 | 0.0193 | 1.1845 | 1.1337 | 123 |
| BNT | 0.0259 | 0.0238 | 41.6469 | 4.8713 | 123 |
| RLC | 0.0188 | 0.0187 | -1.3771 | 0.2690 | 123 |
| MANA | 0.0076 | 0.0131 | 1.1518 | 1.4758 | 123 |
| SNT | 0.0085 | 0.0161 | 13.9888 | 2.9694 | 123 |
| XVG | 0.0112 | 0.0143 | 0.2441 | 0.9397 | 123 |
| IOST | 0.0148 | 0.0262 | 38.9768 | 4.9775 | 123 |
| BTS | 0.0028 | 0.0086 | 16.9119 | 3.7420 | 123 |
| KMD | 0.0155 | 0.0169 | -1.4218 | 0.3895 | 123 |
| STEEM | 0.0111 | 0.0221 | 20.7622 | 3.7033 | 123 |
| MCO | 0.0167 | 0.0453 | 86.0808 | 8.6104 | 123 |
| XZC | 0.0203 | 0.0180 | -0.1232 | 0.4710 | 123 |
| ELF | 0.0106 | 0.0160 | 1.5451 | 1.4313 | 123 |
| ARK | 0.0129 | 0.0216 | 14.8156 | 3.0419 | 123 |
| STRAT | 0.0095 | 0.0153 | 2.6678 | 1.6833 | 123 |
| AION | 0.0166 | 0.0318 | 31.2232 | 4.5910 | 123 |
| STORJ | 0.0160 | 0.0174 | -1.2784 | 0.4112 | 123 |
| WTC | 0.0140 | 0.0202 | 0.2804 | 1.1729 | 123 |
| ENG | 0.0103 | 0.0144 | -0.4701 | 0.9530 | 123 |
| POWR | 0.0108 | 0.0183 | 9.7389 | 2.5501 | 123 |
| NULS | 0.0176 | 0.0205 | 0.2884 | 0.9173 | 123 |
| RCN | 0.0121 | 0.0183 | 0.5077 | 1.2585 | 123 |
| AST | 0.0134 | 0.0195 | 1.7322 | 1.3491 | 123 |
| FUN | 0.0128 | 0.0169 | -0.8010 | 0.8269 | 123 |
| REQ | 0.0151 | 0.0280 | 33.4198 | 4.6754 | 123 |
