# Supplementary material for: Jumps and Cojumps analyses of major and minor cryptocurrencies
Source: PLoS One. 2021 Feb 3;16(2):e0245744. doi: 10.1371/journal.pone.0245744 (PMC7857619; doi:10.1371/journal.pone.0245744)
Supplement: S5 Table — (DOCX) [file pone.0245744.s005.docx]

**S5 Table. Jumps Parameter Estimates**

Table E presents parameter estimates of jump intensity (λ) which is the proportion of trading days with significant jumps, absolute (or unsigned) jump mean (γ*), absolute (or unsigned) jump standard deviation (δ*), (signed) jump mean (γ) which is the mean of the (square rooted) jump variation, (signed) jump standard deviation (δ) which is the standard deviation of the (square rooted) jump variation, and jump contribution $\left( \sum_{t=1}^{T} \sqrt{{JV}_{t,\alpha}\left( \theta\right)} \right)/\left( \sum_{t=1}^{T} \sqrt{{RV}_{t,\alpha}\left( \theta\right)} \right)$ for the major cryptocurrencies and the minor cryptocurrencies. Table A in the appendix presents the list of cryptocurrencies (symbols) considered in this paper as well as their full name and the associated market capitalization according to CoinMarketCap (accessed on June, 2020).

| **SET INDEX** | **λ** | **γ*** | **δ*** | **γ** | **δ** | **Jump Con** |
| --- | --- | --- | --- | --- | --- | --- |
| **SET100** | 0.1545 | 0.0005 | 0.0015 | -0.0004 | 0.0016 | 0.0658 |
| **Cryptocurrencies Ranked by Market Capitalization** | | | | | | |
| **Cryptocurrency** | **λ** | **γ*** | **δ*** | **γ** | **δ** | **Jump Con** |
| BTC | 0.3171 | 0.0062 | 0.0103 | 0.0014 | 0.0120 | 0.1600 |
| ETH | 0.2276 | 0.0023 | 0.0045 | -0.0008 | 0.0050 | 0.0902 |
| XRP | 0.1301 | 0.0015 | 0.0039 | -0.0001 | 0.0042 | 0.0425 |
| LINK | 0.3902 | 0.0113 | 0.0152 | 0.0000 | 0.0190 | 0.1567 |
| LTC | 0.2520 | 0.0035 | 0.0066 | 0.0011 | 0.0074 | 0.1133 |
| ADA | 0.2439 | 0.0046 | 0.0096 | 0.0024 | 0.0104 | 0.0994 |
| EOS | 0.2439 | 0.0057 | 0.0109 | 0.0020 | 0.0121 | 0.1108 |
| BNB | 0.1545 | 0.0028 | 0.0069 | 0.0007 | 0.0074 | 0.0586 |
| XLM | 0.2520 | 0.0048 | 0.0097 | 0.0003 | 0.0109 | 0.1090 |
| TRX | 0.2764 | 0.0058 | 0.0101 | -0.0005 | 0.0117 | 0.0992 |
| XMR | 0.3008 | 0.0064 | 0.0106 | 0.0009 | 0.0124 | 0.1466 |
| NEO | 0.2195 | 0.0040 | 0.0087 | 0.0005 | 0.0096 | 0.0861 |
| IOTA | 0.3171 | 0.0081 | 0.0161 | 0.0007 | 0.0180 | 0.1420 |
| DASH | 0.3252 | 0.0061 | 0.0102 | 0.0014 | 0.0118 | 0.1452 |
| ETC | 0.1870 | 0.0044 | 0.0114 | 0.0011 | 0.0121 | 0.0967 |
| ZEC | 0.2602 | 0.0062 | 0.0117 | 0.0001 | 0.0133 | 0.1175 |
| LEND | 0.4309 | 0.0164 | 0.0202 | -0.0037 | 0.0258 | 0.1942 |
| BAT | 0.2439 | 0.0073 | 0.0138 | -0.0016 | 0.0156 | 0.0972 |
| WAVES | 0.2927 | 0.0066 | 0.0114 | -0.0009 | 0.0132 | 0.1336 |
| ZRX | 0.2439 | 0.0060 | 0.0111 | 0.0003 | 0.0126 | 0.0792 |
| OMG | 0.2846 | 0.0078 | 0.0159 | -0.0012 | 0.0177 | 0.1461 |
| KNC | 0.4634 | 0.0145 | 0.0243 | 0.0012 | 0.0283 | 0.2033 |
| QTUM | 0.1463 | 0.0031 | 0.0078 | 0.0011 | 0.0083 | 0.0611 |
| ICX | 0.1951 | 0.0056 | 0.0132 | 0.0019 | 0.0142 | 0.0826 |
| LSK | 0.3008 | 0.0067 | 0.0126 | 0.0002 | 0.0143 | 0.1281 |
| LRC | 0.3740 | 0.0132 | 0.0280 | 0.0042 | 0.0307 | 0.1749 |
| BTG | 0.2033 | 0.0051 | 0.0110 | -0.0003 | 0.0122 | 0.0912 |
| NANO | 0.2358 | 0.0069 | 0.0139 | 0.0003 | 0.0155 | 0.0945 |
| ENJ | 0.3496 | 0.0107 | 0.0162 | 0.0000 | 0.0194 | 0.1501 |
| BCD | 0.4309 | 0.0149 | 0.0193 | -0.0013 | 0.0244 | 0.1651 |
| BNT | 0.7724 | 0.0259 | 0.0238 | -0.0020 | 0.0352 | 0.4359 |
| RLC | 0.5447 | 0.0188 | 0.0187 | 0.0025 | 0.0265 | 0.2229 |
| MANA | 0.2764 | 0.0076 | 0.0131 | 0.0005 | 0.0151 | 0.0961 |
| SNT | 0.3008 | 0.0085 | 0.0161 | 0.0040 | 0.0177 | 0.1472 |
| XVG | 0.4228 | 0.0112 | 0.0143 | -0.0003 | 0.0182 | 0.1487 |
| IOST | 0.3984 | 0.0148 | 0.0262 | 0.0037 | 0.0299 | 0.1721 |
| BTS | 0.1138 | 0.0028 | 0.0086 | -0.0001 | 0.0090 | 0.0500 |
| KMD | 0.4878 | 0.0155 | 0.0169 | 0.0040 | 0.0227 | 0.2062 |
| STEEM | 0.3171 | 0.0111 | 0.0221 | 0.0067 | 0.0239 | 0.1480 |
| MCO | 0.4228 | 0.0167 | 0.0453 | 0.0044 | 0.0481 | 0.2238 |
| XZC | 0.6423 | 0.0203 | 0.0180 | 0.0026 | 0.0271 | 0.2974 |
| ELF | 0.3659 | 0.0106 | 0.0160 | 0.0030 | 0.0190 | 0.1527 |
| ARK | 0.3740 | 0.0129 | 0.0216 | -0.0018 | 0.0251 | 0.1719 |
| STRAT | 0.3415 | 0.0095 | 0.0153 | 0.0025 | 0.0179 | 0.1701 |
| AION | 0.3740 | 0.0166 | 0.0318 | 0.0000 | 0.0359 | 0.1962 |
| STORJ | 0.4959 | 0.0160 | 0.0174 | -0.0007 | 0.0236 | 0.2180 |
| WTC | 0.3740 | 0.0140 | 0.0202 | -0.0016 | 0.0246 | 0.1711 |
| ENG | 0.3659 | 0.0103 | 0.0144 | -0.0011 | 0.0177 | 0.1459 |
| POWR | 0.3496 | 0.0108 | 0.0183 | 0.0028 | 0.0211 | 0.1556 |
| NULS | 0.4959 | 0.0176 | 0.0205 | -0.0006 | 0.0270 | 0.2249 |
| RCN | 0.3496 | 0.0121 | 0.0183 | 0.0014 | 0.0220 | 0.1699 |
| AST | 0.3659 | 0.0134 | 0.0195 | 0.0011 | 0.0236 | 0.1420 |
| FUN | 0.3984 | 0.0128 | 0.0169 | -0.0061 | 0.0203 | 0.1675 |
| REQ | 0.3902 | 0.0151 | 0.0280 | 0.0027 | 0.0317 | 0.1841 |
