# Supplementary material for: Jumps and Cojumps analyses of major and minor cryptocurrencies
Source: PLoS One. 2021 Feb 3;16(2):e0245744. doi: 10.1371/journal.pone.0245744 (PMC7857619; doi:10.1371/journal.pone.0245744)
Supplement: S6 Table — (DOCX) [file pone.0245744.s006.docx]

**S6 Table. Cojumps Parameter Estimates**

Table F presents parameter estimates of cojump intensity (φ) which is defined as the proportion of trading days with simultaneous (significant) jumps between a pair of testing currency with respect to SET100 index, positive cojump intensity (φ_p_) which is the proportion of days that jumps of both assets are (signed as) positive, negative cojump intensity (φ_n_) which is the proportion of days that jumps of both assets are (signed as) negative, opposed jumps where currency jumps are negative (β_p_), and opposed jumps where currency jumps are positive (β_n_) for the major cryptocurrencies and the minor cryptocurrencies. Table A in the appendix presents the list of cryptocurrencies (symbols) considered in this paper as well as their full name and the associated market capitalization according to CoinMarketCap (accessed on June, 2020).

| **Cryptocurrencies Ranked by Market Capitalization** | | | | | |
| --- | --- | --- | --- | --- | --- |
| **Cryptocurrency** | **φ** | **φ_p_** | **φ_n_** | **β_p_** | **β_n_** |
| BTC | 0.0325 | 0.0081 | 0.0081 | 0.0081 | 0.0081 |
| ETH | 0.0163 | 0.0081 | 0.0000 | 0.0081 | 0.0000 |
| XRP | 0.0325 | 0.0000 | 0.0163 | 0.0163 | 0.0000 |
| LINK | 0.0244 | 0.0000 | 0.0081 | 0.0081 | 0.0081 |
| LTC | 0.0325 | 0.0081 | 0.0081 | 0.0163 | 0.0000 |
| ADA | 0.0325 | 0.0000 | 0.0163 | 0.0081 | 0.0081 |
| EOS | 0.0325 | 0.0081 | 0.0163 | 0.0081 | 0.0000 |
| BNB | 0.0407 | 0.0163 | 0.0081 | 0.0081 | 0.0081 |
| XLM | 0.0163 | 0.0000 | 0.0081 | 0.0000 | 0.0081 |
| TRX | 0.0325 | 0.0000 | 0.0163 | 0.0081 | 0.0081 |
| XMR | 0.0407 | 0.0000 | 0.0081 | 0.0163 | 0.0163 |
| NEO | 0.0407 | 0.0081 | 0.0081 | 0.0163 | 0.0081 |
| IOTA | 0.0488 | 0.0081 | 0.0163 | 0.0163 | 0.0081 |
| DASH | 0.0813 | 0.0081 | 0.0244 | 0.0325 | 0.0163 |
| ETC | 0.0488 | 0.0081 | 0.0407 | 0.0000 | 0.0000 |
| ZEC | 0.0407 | 0.0081 | 0.0081 | 0.0081 | 0.0163 |
| LEND | 0.0650 | 0.0000 | 0.0325 | 0.0163 | 0.0163 |
| BAT | 0.0488 | 0.0000 | 0.0244 | 0.0244 | 0.0000 |
| WAVES | 0.0488 | 0.0325 | 0.0163 | 0.0000 | 0.0000 |
| ZRX | 0.0244 | 0.0081 | 0.0081 | 0.0000 | 0.0081 |
| OMG | 0.0407 | 0.0000 | 0.0244 | 0.0081 | 0.0081 |
| KNC | 0.0650 | 0.0081 | 0.0163 | 0.0244 | 0.0163 |
| QTUM | 0.0244 | 0.0244 | 0.0000 | 0.0000 | 0.0000 |
| ICX | 0.0244 | 0.0163 | 0.0081 | 0.0000 | 0.0000 |
| LSK | 0.0163 | 0.0081 | 0.0000 | 0.0081 | 0.0000 |
| LRC | 0.0488 | 0.0081 | 0.0244 | 0.0163 | 0.0000 |
| BTG | 0.0244 | 0.0000 | 0.0081 | 0.0081 | 0.0081 |
| NANO | 0.0407 | 0.0081 | 0.0244 | 0.0081 | 0.0000 |
| ENJ | 0.0325 | 0.0081 | 0.0081 | 0.0081 | 0.0081 |
| BCD | 0.0569 | 0.0081 | 0.0244 | 0.0081 | 0.0163 |
| BNT | 0.1057 | 0.0244 | 0.0163 | 0.0488 | 0.0163 |
| RLC | 0.0894 | 0.0163 | 0.0325 | 0.0325 | 0.0081 |
| MANA | 0.0813 | 0.0163 | 0.0407 | 0.0081 | 0.0163 |
| SNT | 0.0325 | 0.0081 | 0.0163 | 0.0081 | 0.0000 |
| XVG | 0.0650 | 0.0163 | 0.0244 | 0.0163 | 0.0081 |
| IOST | 0.0894 | 0.0244 | 0.0244 | 0.0325 | 0.0081 |
| BTS | 0.0407 | 0.0163 | 0.0081 | 0.0163 | 0.0000 |
| KMD | 0.0894 | 0.0244 | 0.0407 | 0.0163 | 0.0081 |
| STEEM | 0.0732 | 0.0163 | 0.0081 | 0.0407 | 0.0081 |
| MCO | 0.0488 | 0.0081 | 0.0244 | 0.0081 | 0.0081 |
| XZC | 0.0976 | 0.0244 | 0.0325 | 0.0407 | 0.0000 |
| ELF | 0.0407 | 0.0000 | 0.0163 | 0.0163 | 0.0081 |
| ARK | 0.0813 | 0.0163 | 0.0325 | 0.0163 | 0.0163 |
| STRAT | 0.0244 | 0.0081 | 0.0000 | 0.0081 | 0.0081 |
| AION | 0.0813 | 0.0081 | 0.0488 | 0.0163 | 0.0081 |
| STORJ | 0.0650 | 0.0000 | 0.0163 | 0.0325 | 0.0163 |
| WTC | 0.0407 | 0.0081 | 0.0244 | 0.0000 | 0.0081 |
| ENG | 0.0407 | 0.0081 | 0.0325 | 0.0000 | 0.0000 |
| POWR | 0.0244 | 0.0000 | 0.0000 | 0.0163 | 0.0081 |
| NULS | 0.0569 | 0.0163 | 0.0244 | 0.0081 | 0.0081 |
| RCN | 0.0569 | 0.0081 | 0.0081 | 0.0163 | 0.0244 |
| AST | 0.0407 | 0.0163 | 0.0163 | 0.0081 | 0.0000 |
| FUN | 0.0813 | 0.0000 | 0.0407 | 0.0163 | 0.0244 |
| REQ | 0.0650 | 0.0081 | 0.0244 | 0.0163 | 0.0163 |
