# Supplementary material for: Jumps and Cojumps analyses of major and minor cryptocurrencies
Source: PLoS One. 2021 Feb 3;16(2):e0245744. doi: 10.1371/journal.pone.0245744 (PMC7857619; doi:10.1371/journal.pone.0245744)
Supplement: Attachment — Submitted filename: Response to Key comments from Referees.docx [file pone.0245744.s007.docx]

**Response to Reviewers**

We would like to take this opportunity to thank the reviewers for their valuable suggestions that have helped to enhance the quality of the paper, entitled “Jumps and Cojumps Analyses of Major and Minor Cryptocurrencies”. We have revised the paper accordingly. Below, we list the reviewer’s comments in bold and italics, followed by our responses to each comment.

**Reviewer 1**

1) ***“The existence of jumps has been confirmed by Scaillet et al. [13] for the Bitcoin, and in Luo, M., Kontosakos, V. E., Pantelous, A. A., & Zhou, J. (2019). Cryptocurrencies: Dust in the wind?. Physica A: Statistical Mechanics and its Applications, 525, 1063-1079, The authors provided strong statistical evidence to claim that the prices of cryptocurrencies exhibit positive or negative jumps. They also show that the mass-size distribution of aeolian dust particles provide an excellent model. In other words, the generalized hyperbolic (GH) distribution of Barndorff-Nielsen, O. (1977). Exponentially decreasing distributions for the logarithm of particle size. Proceedings of the Royal Society of London. A. Mathematical and Physical Sciences, 353(1674), 401-419, offers a significantly better fit compared to the normal distribution for modelling daily log-returns of cryptocurrencies.”***

We thank reviewer for this invaluable comment. We have already discussed and cited Scaillet et al. [13] in our paper. For Luo et al. (2019), we would like to thank the reviewer for pointing this paper out to us in which we have discussed and cited this paper in our Section 1 (introduction). In particular, Luo et al. (2019) used the generalized hyperbolic distribution rather than the log-normal distribution in detecting jumps. Also, Luo et al. (2019) examined four cryptocurrencies (Bitcoin, Ethereum, Ripple and Bitcoin cash). From this paper, the reviewer suggested that we should consider using the method of Luo et al. (2019).

We view that the method of Luo et al. (2019) is interesting. However, we believe that using this method is beyond the scope of this paper as the method of Barndorff-Nielsen and Shephard (2004) is sufficient to achieve the objective of this paper from the following reasons. First, as our goal is to demonstrate the differences in the characteristics of jumps and cojumps of major and minor cryptocurrencies, the method of Barndorff-Nielsen and Shephard (2004) is sufficient and does indeed able to demonstrate these differences as we have documented in the paper. The method of Luo et al. (2019) could be interesting for future study that need to model the characteristics of jumps and cojumps of cryptocurrencies more precisely. Second, the method Barndorff-Nielsen and Shephard (2004) is widely used (if not the most widely used) in the finance literature for detecting jumps. Therefore, it would serve our purpose in demonstrating the differences in the characteristics of jumps and cojumps of major and minor cryptocurrencies very well as we used the widely known and acceptable method.

***2)“Based on the above, and considering that there are thousands of really minor cryptocurrencies (>2000), such as MERI (~$285k) or BERRY (~$247k) or TEAM (~$107), just to name a few. You just considered OmiseGo, Zcoin, Everex and Average which have market cap >= $40m. Indeed, the chosen cryptocurrencies are by far smaller than Bitcoin, Ethereum (but I would not consider them as minor), the paper does not provide something really novel and interesting. It is just case study among so many others exist in this field.”***

This point is of great value and we thank the reviewer for pointing this out. We agree with the reviewer that there are many cryptocurrencies in the market. However, our paper covers more cryptocurrencies than existing papers in the literature. For example, Chaim and Laurini (2018) and Scaillet et al.(2020) considered only Bitcoin while Luo et al. (2019) examined only Bitcoin, Ethereum, Ripple and Bitcoin cash. One exception is Bouri et al. (2020) who examined 12 cryptocurrencies which are Bitcoin, Bitshares, Bytecoin, Dash, Digibyte, Dogecoin, Ethereum, Litecoin, Monero, Nem, Ripple and Stellar. However, Bouri et al. (2020) only focuses on detecting the presence of jumps and cojumps in these cryptocurrencies without examining and emphasizing the differences in the characteristics of jumps and cojumps of major and minor cryptocurrencies.

In our revision, we strengthen the contribution of this paper by increasing the number of cryptocurrencies (both major and minor cryptocurrencies) covered in the paper. In total, we examine 54 cryptocurrencies. To the best of our knowledge, this is the most extensive study of cryptocurrencies in the literature. Table A in the appendix presents the list of cryptocurrencies (symbols) considered in this paper as well as their full name and the associated market capitalization according to CoinMarketCap (accessed on June, 2020). Among these cryptocurrencies, 3 cryptocurrencies have market capitalization more than 10 Billion USD, 10 cryptocurrencies have market capitalization between than 1 Billion USD and 10 Billion USD, 23 cryptocurrencies have market capitalization between than 100 Million USD and 1 Billion USD and 18 cryptocurrencies have market capitalization less than 100 Million USD. We have discussed and incorporated this throughout the revised paper (for example, in Section 1 (introduction), Section 3 (data), Section 4 (Empirical Findings), Section 5 (conclusion), Table 1, Table 2, Table A, Table B, Table C, Table D, Table E, and Table F, respectively).

**Reviewer 2**

***1) “Cryptocurrencies are traded 24 hours a day, whereas Thailand Stock Exchange is only open during certain hours. Therefore, how do you match data from both cryptocurrencies and stock exchange? From a technical point of view, cryptos information flow is continuous, but SET100 will have some periods without trading. Probably the authors should comment on this aspect.”***

We thank the reviewer for pointing this out to us. In the revised paper, we have added the information and discussion about this point in Section 3 (data). In particular, for detecting jumps and cojumps of major and minor cryptocurrencies and the Thai stock market index, we revise our analysis to exclude weekends and holidays in the Thai stock market.

***2) “The authors should consider some related literature, regarding co-movements. For example: Eng-Tuck Cheah, Tapas Mishra, Mamata Parhi, Zhuang Zhang, Long Memory Interdependency and Inefficiency in Bitcoin Markets, Economics Letters, Volume 167, 2018,Pages 18-25, https://doi.org/10.1016/j.econlet.2018.02.010. Paraskevi Katsiampa, Volatility co-movement between Bitcoin and Ether, Finance Research Letters, Volume 30,2019,Pages 221-227, https://doi.org/10.1016/j.frl.2018.10.005.”***

We thank the reviewer for this great comment, In the revised paper, we have added the papers about co-movement and discussion them in Section 1 (introduction, footnote #2).

***3)“In the empirical findings section, the authors say: "we found that the minor cryptocurrencies have the highest percentage of days with significant jumps". In this aspect,*** ***Bariviera, A. F. (2020). One model is not enough: heterogeneity in cryptocurrencies’ multifractal profiles. Finance Research Letters, (June), 101649. https://doi.org/10.1016/j.frl.2020.101649 already found that small cryptos are prone to sudden jumps. Therefore it could be interesting to link the authors' findings with (probably) the multifractal behavior of small cryptos whose dynamics is more likely to exhibit such jumps.”***

Noted with thanks. In the revised paper, we have linked our finding to Bariviera, A. F. (2020) and other papers about the multifractal behavior in Section 4 (empirical findings).

***4)“The introduction could be slightly expanded, introducing some discussion on the informational efficiency and portfolio topics. Literature on cryptocurrencies is very abundant. You could select 3 o 4 of the most cited papers in this area. This last recommendation is because the authors discuss (in conclusions) the importance of their findings for regulatory purposes. However they do not comment on the effect that their findings could have for portfolio analysis (portfolios constituted by cryptos only, or cryptos and SET100 stocks).”***

We thank the reviewer for this great comment. In our revision, we have added a discussion about informational efficiency and portfolio theory in Section 1 (introduction). We have also added more references about cryptocurrency that could provide a better understanding and discussion about informational efficiency and portfolio theory for the case of cryptocurrency.

**Reviewer 3**

***1) “Page 2 – “However, very few studies have examined the presence of jumps and cojumps in cryptocurrencies.” Add references on jumps and especially co-jumps in cryptocurrencies. A direct modeling of joint jumps in the mean and conditional variance for cryptocurrencies can be found at Chaim and Laurini (2019), Nonlinear dependence in cryptocurrency markets, The North American Journal of Economics and Finance, Volume 48, 2019, Pages 32-47.”***

We thank the reviewer for pointing this out. In the revision, we have added the reference suggested by the reviewer and discuss about this paper in the Section 1 (Introduction).

2) ***“Typo in title of Table 1 – ‘Jumps and Cojumps Paramter Estimates’” and***
3) ***“Full reference for the cited working papers in the references’”***

Noted with thanks.

***4) “Since PLOS ONE is a general public journal, it would be important to post a brief discussion on the importance of jumps and co-jumps in terms of asset allocation and risk management.”***

Noted with thanks. We have added a brief discussion on the importance of jumps and cojumps for asset allocation and risk management in the Section 1 (Introduction).

Respectfully yours,

Corresponding Author of the paper entitled “Jumps and Cojumps Analyses of Major and Minor Cryptocurrencies”
